# From Evidence to Implementation: FIB-4-Based Risk Stratification for Advanced Liver Disease in Saudi Primary Care

**DOI:** 10.3390/medicina62102037

**Published:** 2026-10-21

**Authors:** Yusef Muhana Alenezi

**Affiliations:** Family and Community Department, Faculty of Medicine, Northern Border University, Arar 91431, Saudi Arabia; dr.yusefmd@gmail.com; Tel.: +966-146615660

**Keywords:** FIB-4, MASLD, liver fibrosis, primary care, Saudi Arabia, non-invasive tests, case-finding, risk stratification

## Abstract

Metabolic dysfunction-associated steatotic liver disease (MASLD) is increasingly common and poses a growing challenge for health systems, because most affected people are managed outside specialist liver services. The clinically important task is not simply to identify hepatic steatosis, but to find the smaller subgroup with clinically significant fibrosis who face the greatest risk of liver-related complications and who may now be eligible for liver-directed pharmacotherapy. The fibrosis-4 index (FIB-4), calculated from age, aspartate aminotransferase, alanine aminotransferase and platelet count, has emerged as a pragmatic first-line tool for fibrosis risk stratification. Contemporary guidance recommends a stepwise approach in at-risk populations, with FIB-4 followed by second-line non-invasive testing, most commonly vibration-controlled transient elastography (VCTE) or the Enhanced Liver Fibrosis (ELF) test. The September 2026 Global NASH/MASH Council consensus consolidates this fibrosis-centred, non-invasive-test-driven model further and links second-line test thresholds to treatment initiation and response monitoring in non-cirrhotic MASH with F2–F3 fibrosis. Saudi Arabia is a particularly relevant setting given its substantial cardiometabolic burden and emerging evidence of a high MASLD prevalence. Recent Saudi data include a multicentre primary-care cohort reporting an age- and sex-adjusted MASLD prevalence of 34.2%, a real-world primary-care study demonstrating intermediate or high FIB-4 risk among metabolically high-risk patients despite normal liver enzymes, and a Saudi consensus incorporating FIB-4 into local risk stratification. Important implementation questions remain, however, including target populations, age-related interpretation, laboratory readiness, second-line testing capacity, referral pathways, treatment access, repeat assessment, electronic health-record integration and cost-effectiveness. This narrative review traces the evolution of FIB-4 from a prognostic marker to a primary-care triage tool, critically examines contemporary international and Saudi evidence, and proposes a context-sensitive framework and staged research agenda for FIB-4-based risk stratification in Saudi primary care.

## 1. Introduction

Metabolic dysfunction-associated steatotic liver disease (MASLD), the term that replaced non-alcoholic fatty liver disease (NAFLD) after the 2023 multi-society nomenclature process [1], is now the most common form of chronic liver disease worldwide [1,2]. The change in terminology is more than semantic: it places cardiometabolic dysfunction at the centre of the disease construct and aligns liver disease more closely with the populations already managed in primary care, diabetes, obesity and cardiovascular-risk services. Earlier NAFLD evidence remains largely applicable, since there is substantial overlap between the populations captured by the old and new definitions [1,3].

The scale of the problem is considerable. A global systematic review estimated NAFLD prevalence at approximately 30%, with a marked increase over time and a particularly high burden in the Middle East and North Africa [4], and more recent modelling confirms that this burden has continued to grow over the past three decades [5]. Prevalence of steatosis alone, however, does not define the clinical burden that should drive referral and specialist resource use. Fibrosis stage is a far stronger determinant of liver-related outcomes: meta-analytic data show a stepwise increase in all-cause mortality as fibrosis advances, with an even steeper increase in liver-related mortality [6]. This shifts the clinical question from ‘who has fatty liver?’ to ‘who is at meaningful risk of progressive liver disease?’

Primary care sits at the centre of that question. Patients with type 2 diabetes, obesity, hypertension and dyslipidaemia are frequently seen in primary care long before liver disease becomes symptomatic; liver enzymes may remain normal despite clinically important fibrosis, and universal referral for imaging or specialist assessment would be impractical in a high-prevalence condition [7,8]. What primary care needs instead is a low-cost first-line method that can exclude advanced fibrosis in most patients and identify a smaller group for second-line assessment.

The fibrosis-4 index (FIB-4) has increasingly filled this role. Originally developed in patients with HIV/HCV coinfection, it uses age, aspartate aminotransferase (AST), alanine aminotransferase (ALT) and platelet count, all routinely available variables [9]. FIB-4 is not a definitive diagnostic test for advanced fibrosis; its value in primary care lies primarily in triage. A low score identifies patients at low probability of advanced fibrosis, while an indeterminate or high score triggers further assessment. Contemporary AASLD and EASL-EASD-EASO guidance, together with the 2026 American Gastroenterological Association (AGA) clinical care pathway, all embed FIB-4 within a sequential non-invasive testing strategy [7,8,10].

Saudi Arabia provides a timely setting in which to examine this transition from evidence to implementation. A 2023 Saudi systematic review and meta-analysis found substantial heterogeneity in NAFLD prevalence estimates and highlighted the need for better population-based evidence [11]. The evidence base has since moved on. A 2026 prospective population-based study spanning seven primary-care centres reported an age- and sex-adjusted MASLD prevalence of 34.2%, with significant fibrosis present in 4.3% of participants with MASLD [12]. A Saudi Delphi consensus has incorporated FIB-4 and other non-invasive tests into local recommendations [13], and a 2026 real-world primary-care study has demonstrated the feasibility of FIB-4-based risk stratification among metabolically high-risk adults with normal liver enzymes [14].

The clinical significance of early fibrosis identification has also changed. The 2026 Global NASH/MASH Council consensus, developed after an updated literature review and a Delphi process involving 40 international experts, retained FIB-4 as the preferred first-line risk-assessment tool, followed by VCTE or ELF, but extended the pathway beyond risk prediction to treatment selection and objective response monitoring [15]. This evolution matters for primary care: identifying F2–F3 fibrosis is now increasingly actionable rather than merely prognostic, which raises the cost of missed case-finding and makes efficient referral-pathway design more urgent.

This review therefore focuses on the implementation problem rather than offering another general description of FIB-4. It aims to: (1) summarise why fibrosis risk, rather than steatosis alone, should drive case-finding; (2) critically review the role and limitations of FIB-4 as a first-line primary-care tool; (3) examine the evolution of sequential non-invasive testing pathways; (4) synthesise the emerging Saudi evidence; and (5) propose a context-sensitive framework and research agenda for FIB-4-based risk stratification in Saudi primary care.

## 2. Materials and Methods

This narrative review was informed by a structured search of the biomedical literature. PubMed/MEDLINE and major professional-society guideline repositories were searched for literature relevant to MASLD/NAFLD, chronic liver disease, liver fibrosis, FIB-4, APRI, non-invasive liver tests, primary care, case-finding, risk stratification, Saudi Arabia and the Gulf region. The review prioritised clinical practice guidelines and care pathways, systematic reviews, longitudinal and diagnostic studies, primary-care implementation studies, health-economic analyses and Saudi empirical studies. Foundational publications on the development and validation of FIB-4 were retained where necessary to explain the evolution of the evidence.

Earlier literature was supplemented by reference screening of relevant publications identified during prior work on non-invasive biomarkers and advanced liver disease. The literature search was updated through September 2026, with particular attention to studies published after 2023, since the MASLD nomenclature, international clinical pathways and Saudi evidence all changed substantially over this period. Because the purpose of the review was interpretive and implementation-focused rather than to estimate a pooled effect, formal meta-analysis and systematic risk-of-bias scoring were not undertaken; reporting instead followed the general principles set out in the SANRA (Scale for the Assessment of Narrative Review Articles) framework [16]. The distinction between empirical evidence, guideline recommendations and the implementation framework proposed in this review is maintained throughout.

## 3. Results

### 3.1. From MASLD Burden to Fibrosis Risk: Why the Saudi Context Matters

Saudi Arabia has undergone a rapid epidemiological and lifestyle transition, with a high burden of obesity, type 2 diabetes and related cardiometabolic disease, all major drivers of MASLD. Earlier Saudi studies varied widely in setting, population and diagnostic method, producing substantial heterogeneity in prevalence estimates. The 2023 systematic review by Alenezi and colleagues pooled disease-unrestricted Saudi studies and highlighted both a substantial burden and the limitations of the available evidence [11]. That heterogeneity was itself clinically important: a single historical prevalence estimate should not be treated as a precise national figure.

A 2026 prospective cohort study by Alqahtani and colleagues materially advances this evidence base. In a cross-sectional analysis nested within a prospective population-based cohort, 2979 adults were recruited across seven Saudi primary-care centres between March 2024 and June 2025 and underwent clinical assessment, laboratory testing and vibration-controlled transient elastography (VCTE). MASLD was present in 1111 participants, corresponding to a crude prevalence of 37.3% and an age- and sex-adjusted prevalence of 34.2%. Among participants with MASLD, 4.3% met the study definition of significant fibrosis (liver stiffness measurement ≥ 8 kPa); diabetes, male sex and waist circumference were independently associated with significant fibrosis [12].

These findings carry two implications. First, they confirm that MASLD is too common for specialist-led assessment of every affected individual to be realistic. Second, the comparatively small proportion with significant fibrosis reinforces the case for triage over blanket screening: a pathway that safely retains low-risk patients in primary care, while escalating a smaller group for elastography or specialist assessment, is more useful than one designed simply to label steatosis.

Saudi prevalence data should nonetheless be interpreted carefully. This cohort study represents a major improvement in design and phenotyping, but it does not by itself establish the optimal national screening or case-finding strategy. The distribution and performance of FIB-4, the proportion of patients requiring second-line testing, regional variation in access to VCTE, and downstream referral capacity all remain separate implementation questions.

### 3.2. Why Fibrosis, Rather than Steatosis Alone, Should Drive Risk Stratification

MASLD spans a broad spectrum, from steatosis without fibrosis to cirrhosis and its complications. For clarity, this review uses significant fibrosis to refer to histological stage F2 or higher (F2–F4) and advanced fibrosis to refer to stage F3 or higher (F3–F4). These categories overlap but are not interchangeable: F2 is clinically significant without being advanced, while F3–F4 meets both definitions. Advanced liver disease is a broader clinical expression and should not be treated as a synonym for either fibrosis category. The distinction matters because FIB-4 pathways were developed principally to identify patients unlikely to have advanced fibrosis, whereas emerging treatment pathways also seek to identify selected patients with non-cirrhotic F2–F3 MASH [7,8,15].

The emphasis on fibrosis is supported by longitudinal and meta-analytic evidence. Mortality risk increases with fibrosis stage, including a rise in liver-related mortality from F2 onward [6]. Long-term biopsy cohorts have identified fibrosis stage as a stronger predictor of disease-specific mortality than histological activity [17], and the Global-MASLD cohort further supports the prognostic importance of fibrosis [18]. Accordingly, this review distinguishes the broader group with significant fibrosis (F2–F4), relevant to disease progression and treatment assessment, from the subgroup with advanced fibrosis (F3–F4), for whom specialist evaluation and surveillance decisions are especially important [7,8].

Routine liver enzymes cannot reliably perform this task. Aminotransferase concentrations reflect hepatocellular injury and fluctuate; they do not map directly onto fibrosis stage. AASLD guidance explicitly notes that aminotransferases are frequently normal in patients with advanced disease and should not be used in isolation to exclude clinically significant fibrosis [7], a point of particular importance in primary care, where a normal ALT can create false reassurance in patients with substantial metabolic risk.

The practical implication is a shift from liver-enzyme-triggered investigation to risk-based case-finding. Instead of waiting for abnormal biochemistry, clinicians identify individuals with a risk profile, especially type 2 diabetes, obesity with additional metabolic risk, established steatosis or persistently abnormal liver tests and then apply a simple first-line fibrosis assessment. FIB-4 has become the most widely recommended blood-based tool for this purpose.

### 3.3. FIB-4: From Fibrosis Marker to Primary-Care Triage Tool

#### 3.3.1. Development and Rationale

FIB-4 was developed by Sterling and colleagues in 2006 using routinely available variables in patients with HIV/HCV coinfection [9]. The formula is: age (years) × AST (U/L)/[platelet count (10^9^/L) × √ALT (U/L)]. Its appeal lies in its simplicity and its lack of proprietary laboratory components. Although the original derivation population differs from contemporary MASLD cohorts, subsequent validation across a range of liver-disease settings has established FIB-4 as a practical non-invasive fibrosis score, including within a dedicated AASLD guideline on blood-based non-invasive assessment of hepatic fibrosis and steatosis [19].

In primary care, the objective is not to replace elastography or histology with FIB-4. Rather, FIB-4 acts as a gatekeeper: its greatest value is the ability to classify a large proportion of at-risk patients as low probability for advanced fibrosis, thereby concentrating more expensive or less accessible second-line tests on those who actually need them.

#### 3.3.2. Interpretation and Thresholds

Commonly used MASLD pathways define FIB-4 < 1.3 as low risk, 1.3–2.67 as an intermediate or indeterminate range, and >2.67 as high risk [7]. These thresholds are not universal biological boundaries; they are decision thresholds whose performance depends on age, disease prevalence and clinical setting. AASLD recommends an age-adjusted threshold (>2.0) in adults older than 65 years and cautions that FIB-4 has low accuracy in people younger than 35 years [7]. EASL-EASD-EASO similarly recommends a multistep strategy rather than interpreting FIB-4 in isolation [8].

The age component creates an important implementation issue. Older age raises FIB-4 even when AST, ALT and platelets are relatively stable, increasing false-positive classification in older adults. Conversely, younger adults may have deceptively low scores. A Saudi pathway should therefore avoid embedding a single threshold into an electronic system without age-aware interpretation and a clear plan for patients outside the age range in which FIB-4 performs best.

#### 3.3.3. Strengths

FIB-4 has several characteristics that make it attractive for primary care. It uses routinely collected variables; it can be calculated without additional blood sampling when the necessary tests are already available; it has been extensively validated; and it carries minimal direct test cost. It can also be repeated longitudinally. In a low-prevalence primary-care setting, these features support its role as an exclusion and triage tool rather than a stand-alone diagnostic test [7].

An additional advantage is scalability. If AST, ALT and platelet results are available in the electronic health record (EHR), FIB-4 can be calculated automatically without relying on clinician memory, creating the possibility of automated case-finding among predefined high-risk groups. Automation introduces its own challenges; however, defining eligibility, preventing inappropriate calculation during acute illness, applying age-specific interpretation, and ensuring that an elevated result triggers an actionable pathway rather than an isolated alert.

#### 3.3.4. Limitations

FIB-4 has important limitations. It is influenced by age, acute illness, thrombocytopenia unrelated to liver fibrosis, and transient aminotransferase changes. A substantial intermediate zone means many patients require second-line assessment. Its positive predictive value is modest in low-prevalence populations, and its performance may differ in diabetes and other high-risk groups. It should therefore never be presented to clinicians or patients as a diagnosis of advanced fibrosis.

Recent consensus also emphasises that an FIB-4-first strategy should not be applied rigidly across every subgroup. Diagnostic performance may be reduced in patients with complicated type 2 diabetes, for whom VCTE or ELF may reasonably be considered as first-line testing in selected settings [15]. This is especially relevant in Saudi Arabia, where diabetes is likely to account for a substantial proportion of the population entering a fibrosis case-finding pathway. Local validation should therefore examine both the standard FIB-4-first model and whether selected very-high-risk groups benefit from direct second-line non-invasive testing.

A further limitation is data availability. A score that is mathematically simple cannot be calculated if AST, ALT or platelets are missing or were measured at clinically inappropriate intervals. This issue is particularly relevant when FIB-4 is derived retrospectively from routine records, or when laboratory panels differ across primary-care systems. Implementation research should therefore measure not only the distribution of FIB-4 but also the proportion of eligible patients in whom the score can be validly calculated.

### 3.4. FIB-4 Should Be the First Step, Not the Final Test

The strongest contemporary model is sequential testing. EASL-EASD-EASO recommends an established blood-based score such as FIB-4 first, followed by liver elastography when fibrosis remains suspected or in high-risk groups, with ELF available as an alternative second-line blood test [8]. AASLD similarly recommends VCTE or ELF for patients with elevated FIB-4 [7]. More recent evidence has strengthened this architecture: a 2025 New England Journal of Medicine review positioned FIB-4 within contemporary non-invasive fibrosis assessment [20], while a 2025 study demonstrated the prognostic utility of a two-step clinical care pathway in MASLD [21]. September 2026 Global NASH/MASH Council consensus retained FIB-4 as the preferred first-line tool and VCTE or ELF as second-line tests, explicitly prioritising these modalities because they are scalable across primary-care and community settings [15].

The logic is straightforward. FIB-4 is inexpensive and scalable but imperfectly specific. VCTE and ELF are more informative but less suitable as universal first-line tests because of cost, access and service capacity. Sequential testing combines the strengths of both: low-risk patients remain in primary care, patients in the uncertain group undergo a more specific second-line test, and specialist resources are focused on those with persistently elevated risk.

Primary-care implementation studies support this model. A prospective UK pathway using FIB-4 followed by ELF analysed 3012 patients and was associated with a marked reduction in unnecessary referrals while substantially increasing detection of advanced fibrosis and cirrhosis [22]. Economic models have likewise suggested that two-tier strategies using FIB-4 followed by ELF or elastography can reduce unnecessary specialist referrals and improve resource use compared with usual care [23,24,25,26]. A 2025 UK economic evaluation found FIB-4/ELF and FIB-4/transient-elastography pathways cost-saving relative to standard care in a simulated high-risk MASLD primary-care population [25]. Evidence for a FIB-4–VCTE pathway is also available. In a multicentre study of 595 patients across primary and hospital care, a two-step strategy using FIB-4 ≥ 1.30 followed by VCTE increased correct referrals fivefold compared with regular care (95% CI 2.4–10.1) [27]. This supports elastography as an effective second-line option alongside ELF, although local access and referral arrangements will determine which pathway is practical in Saudi settings.

These findings cannot be transferred mechanically to Saudi Arabia, because costs, referral structures, laboratory practice and access to elastography differ. They do, however, provide a strong rationale for evaluating a two-tier Saudi pathway rather than testing a model in which every elevated FIB-4 result is sent directly to hepatology.

### 3.5. Evolution of International Guidance: From Screening Debate to Targeted Case-Finding

International recommendations have converged on targeted case-finding rather than universal population screening [28]. AASLD does not advise general population screening for NAFLD but recommends primary risk assessment with FIB-4 in patients with steatosis or clinically suspected disease on the basis of obesity and metabolic risk factors, with more frequent reassessment in people with prediabetes, type 2 diabetes or multiple metabolic risk factors [7].

The 2024 EASL-EASD-EASO guideline is particularly explicit. It recommends evaluation for MASLD and advanced fibrosis in people with type 2 diabetes, abdominal obesity plus at least one additional metabolic risk factor, or persistently elevated liver enzymes. FIB-4 is recommended as the first step, followed by elastography or, where appropriate, ELF [8]. This formulation is useful for Saudi primary care because it defines a high-risk entry population rather than requiring known steatosis before fibrosis assessment.

The 2026 AGA clinical care pathway reflects a further shift toward operational implementation. It identifies patients at risk for clinically significant fibrosis, emphasises sequential non-invasive testing, and includes implementation as a dedicated component of the pathway [10]. Taken together, these documents support a pragmatic principle: the objective is not to screen every adult for MASLD, but to identify fibrosis risk efficiently in populations where the pre-test probability is sufficiently high to justify assessment. Saudi professional recommendations are also included in Table 1; their implications for local referral practice are considered in Section 3.6.2 [13].

### 3.6. What Do We Now Know in Saudi Arabia?

#### 3.6.1. Epidemiological Evidence

For implementation, the main contribution of recent Saudi epidemiological research is not simply a more reliable estimate of MASLD prevalence. The multicentre primary-care cohort provides a basis for identifying where targeted fibrosis assessment might be most productive and for anticipating demand for VCTE [12]. Its findings suggest that case-finding should be evaluated in clinically defined risk groups rather than applied indiscriminately across all adults. However, the proportion with a raised FIB-4, the number who would proceed to VCTE or ELF, and the subsequent specialist workload cannot be inferred directly from MASLD prevalence or a single liver-stiffness threshold.

Diabetes, male sex and waist circumference were associated with significant fibrosis in the Saudi cohort [12]. These observations can inform the design of local validation studies and help identify subgroups in which missed disease and unnecessary testing should be measured separately. Before national implementation, primary-care datasets should be used to examine FIB-4 calculability, age-specific score distributions, regional access to second-line testing and the proportion of patients completing each stage of the pathway.

#### 3.6.2. Saudi Consensus Recommendations

A modified Delphi consensus published in the Saudi Journal of Gastroenterology developed local recommendations for the early detection, referral and management of MASLD/MASH. The panel included hepatology, gastroenterology, endocrinology and primary-care expertise and reviewed evidence through May 2024 [13]. The recommendations place low-risk patients, including those with FIB-4 < 1.3, in primary-care follow-up, and identify high-risk non-invasive test results as an indication for specialist referral, an important step, because it establishes local professional support for risk-stratified care.

Consensus is not the same as implementation validation, however. A threshold may be clinically reasonable and internationally supported while the consequences of applying it across a national primary-care system remain uncertain. The number of patients generated for VCTE, the availability of second-line testing, regional referral capacity, clinician adherence and patient follow-up all need to be measured empirically.

#### 3.6.3. Emerging Real-World Primary-Care Evidence

A 2026 single-centre retrospective study from Saudi Arabia evaluated 200 adults in primary care with obesity and type 2 diabetes, no known liver disease and normal liver enzymes. FIB-4 classified 123 patients (61.5%) as low risk, 71 (35.5%) as intermediate risk and 6 (3.0%) as high risk; gastroenterology referral was limited [14]. The study is important because it addresses a practical blind spot: metabolically high-risk patients may have normal routine liver biochemistry and might never enter a liver-disease pathway at all.

The study should nonetheless be read as an implementation signal rather than a definitive validation. It was retrospective, single-centre, and selected a highly specific high-risk population. FIB-4 categories were not equivalent to confirmed fibrosis stages, and the study does not establish the national prevalence of advanced fibrosis. Its value lies in demonstrating that routine primary-care data can identify a clinically relevant group who would otherwise be overlooked, and in exposing the gap between risk identification and referral. Table 2 summarizes the key Saudi studies and consensus recommendations discussed above, highlighting their main contributions and limitations for implementing FIB-4-based risk stratification in primary care.

### 3.7. Why Earlier Detection Is More Actionable in 2026: The Therapeutic Shift

The rationale for fibrosis case-finding has changed substantially since the earlier generation of FIB-4 pathways. Historically, the principal benefit of identifying advanced fibrosis was prognostic: stratification, surveillance, management of complications and prioritisation for specialist care. The availability of liver-directed therapy for selected patients with non-cirrhotic MASH and F2–F3 fibrosis means that identification of clinically significant fibrosis can now influence treatment eligibility as well as prognosis [15,28,29]. Here, significant fibrosis includes F2–F4, while advanced fibrosis denotes F3–F4; eligibility for therapies in non-cirrhotic MASH may include selected F2–F3 patients and should not be inferred from FIB-4 alone [15,29,30].

The 2026 global consensus supports consideration of resmetirom or semaglutide in appropriately selected non-cirrhotic patients whose second-line non-invasive tests suggest F2–F3 fibrosis, once cirrhosis has been excluded [15,28,29]. The consensus identifies VCTE liver stiffness values of approximately 10 to <20 kPa and ELF values of approximately 9.2 to <11.3 as clinically actionable ranges, while acknowledging that treatment decisions require the total clinical context and that some thresholds need further prospective validation [15], an approach consistent with evidence that specific VCTE thresholds independently predict liver-related clinical events [31]. These thresholds should not be transferred directly into a Saudi primary-care protocol without local specialist governance and consideration of regulatory approval and access.

Non-invasive tests are also moving from one-time staging tools toward longitudinal markers. The consensus defines a reduction of at least 30% in VCTE-measured liver stiffness, or of at least 0.5 points in ELF, as evidence supporting treatment response, while worsening should be confirmed with a second concordant test before management is changed [15]. This reinforces a broader concept relevant to Saudi implementation: a fibrosis pathway needs continuity from case-finding through confirmation and treatment decision-making to follow-up, rather than ending at the calculation of FIB-4.

These treatment developments do not, in themselves, justify indiscriminate population screening. Instead, they strengthen the case for efficient, targeted case-finding in populations with sufficient pre-test risk. The American Diabetes Association’s 2025 consensus report specifically emphasised screening and early intervention for MASLD in people with diabetes [32]. For Saudi primary care, the practical challenge is therefore to identify a manageable high-risk population and connect first-line FIB-4 to accessible confirmatory testing and specialist decision-making.

### 3.8. The Saudi Implementation Gap

The question for Saudi Arabia is therefore no longer whether fibrosis risk stratification is relevant. The remaining challenge is how to operate it. Several implementation domains require explicit evaluation.

Target population. International guidance supports targeted case-finding, but a Saudi pathway must define who enters it. Type 2 diabetes is the clearest candidate group. Other potential triggers include obesity with additional metabolic risk, documented steatosis, persistent liver-enzyme abnormalities, or combinations of cardiometabolic factors. A pathway that is too broad may overwhelm second-line services; one that is too narrow may miss clinically important disease.

Laboratory readiness. FIB-4 requires contemporaneous age, AST, ALT and platelet data. Primary-care laboratory practice should be audited to determine how often these components are available together in eligible patients. If AST is not routinely included in relevant panels, automated FIB-4 cannot function simply by adding an EHR calculation.

EHR integration. Manual calculation is unlikely to support large-scale adoption. Automated calculation could be triggered when eligible patients have the required laboratory results, and the EHR should present the score together with its interpretation and a next action, since a number without a pathway risks alert fatigue. The system should also suppress or qualify results in settings where FIB-4 is unreliable, such as acute illness, and should apply age-aware interpretation.

Second-line testing capacity. Demand for VCTE or ELF should be estimated using age-aware FIB-4 interpretation, rather than applying ≥1.3 uniformly. In adults aged 35–65 years, FIB-4 ≥1.3 generally warrants further risk assessment; in those older than 65 years, a threshold of >2.0 is commonly used to reduce age-related false positives [7]. FIB-4 performs poorly below age 35, so clinical risk factors and the possible need for direct second-line assessment should take precedence over an apparently reassuring score. Saudi services need data on the expected volume, geographical distribution and waiting time for second-line assessment. Regional models may include hospital-based, hub-and-spoke or selected primary-care VCTE provision.

Referral thresholds and shared care. A moderately elevated FIB-4 should not automatically lead to hepatology referral: second-line VCTE or ELF can refine risk and reduce unnecessary appointments. By contrast, FIB-4 > 2.67, concerning clinical features, persistent aminotransferase elevation or suspected alternative liver disease support direct specialist evaluation, while abnormal or indeterminate second-line findings should trigger referral according to locally agreed thresholds [7,8,13]. The distinction is between testing to resolve uncertainty and referral to investigate a sufficiently high or clinically concerning probability of disease. Responsibility for metabolic management, repeat testing, surveillance and return to primary care should be explicit.

Reassessment intervals. A low FIB-4 score is not a lifetime classification. AASLD suggests more frequent reassessment in people with prediabetes, type 2 diabetes or multiple metabolic risk factors [7], and EASL-EASD-EASO also supports periodic reassessment [8]. Saudi implementation research should evaluate whether these intervals are feasible, and whether repeated FIB-4 measurement meaningfully improves detection in local populations.

Equity and regional access. Implementation should be assessed beyond tertiary centres. A pathway that performs well in a highly resourced integrated system may not produce the same outcomes in geographically dispersed primary-care networks. Regional access to laboratory testing, VCTE, specialist review and follow-up should therefore be treated as an implementation outcome rather than an afterthought.

Health economics. International analyses suggest that sequential non-invasive test strategies can reduce unnecessary referrals and may be cost-saving [23,24,25,26], but Saudi costs, service structures and opportunity costs differ. A local economic evaluation should incorporate the cost of laboratory testing, EHR development, elastography, specialist appointments, false-positive referrals, false-negative disease and downstream complications.

### 3.9. Proposed Saudi Primary-Care Framework

**Figure 1:**
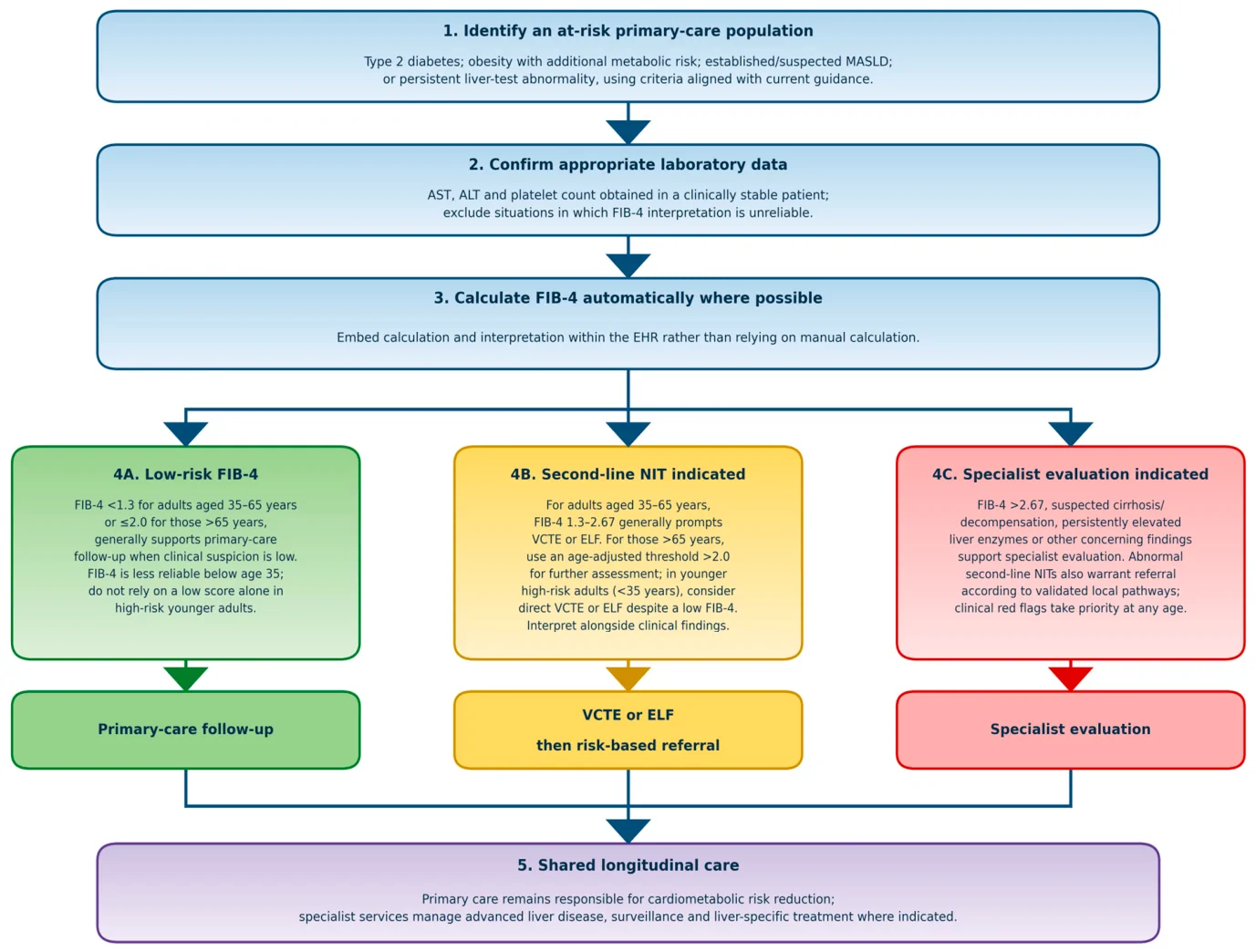
Proposed FIB-4-based triage framework for Saudi primary care. The framework distinguishes second-line non-invasive testing from specialist evaluation and significant fibrosis (F2–F4) from advanced fibrosis (F3–F4), which is a primary target for specialist referral (Section 3.2). For adults aged 35–65 years, FIB-4 < 1.3 generally supports primary-care follow-up, 1.3–2.67 prompts VCTE or ELF assessment, and >2.67 supports specialist evaluation. In adults older than 65 years, a threshold of >2.0 is commonly used to guide further assessment. FIB-4 is less reliable in adults younger than 35 years, for whom clinical risk may justify direct second-line testing. Clinical red flags override score-based triage. These thresholds are intended as risk-stratification tools rather than diagnostic indicators of fibrosis stage. The proposed pathway requires local validation before implementation [7,8,13].

The framework below (Figure 1) is proposed as an implementation and research model, not a validated Saudi national clinical guideline. It separates the need for a second-line non-invasive test from the need for specialist assessment. In clinically stable adults with an interpretable FIB-4, a score below 1.3 generally supports follow-up in primary care, with risk-appropriate reassessment. A score from 1.3 to 2.67 usually calls for VCTE or ELF before deciding on referral; a value above 2.67 warrants consideration of direct specialist referral because the probability of advanced fibrosis is higher [7,8]. For adults older than 65 years, the lower decision threshold is generally adjusted to 2.0; FIB-4 is less reliable below age 35 [7]. These cut-offs indicate triage decisions rather than confirmed histological stages. Following second-line assessment, low-risk findings may support continued primary-care management, whereas elevated or indeterminate findings, or discordance with the clinical picture, require specialist discussion or evaluation according to locally agreed thresholds [7,8,13]. Clinical features suggesting cirrhosis or decompensation, persistently elevated aminotransferases, or concern for another liver disease may justify referral irrespective of FIB-4, without waiting for sequential testing [7]. In selected high-risk patients, direct VCTE or ELF may also be appropriate [15]. The usual 1.3 threshold should not be applied indiscriminately: for adults older than 65 years, >2.0 is the commonly recommended threshold for further assessment, whereas FIB-4 is less reliable in those younger than 35 years and clinical risk may justify direct VCTE or ELF [7]. These age considerations should be implemented alongside, not instead of, assessment for clinical red flags.

### 3.10. Research Priorities: A Saudi Programme from Validation to Effectiveness

A useful narrative review should not end by calling generically for ‘more research’. The current evidence allows a more structured Saudi research programme.

Phase 1: Define the eligible population and baseline distribution. Large routine-data studies should estimate how many Saudi primary-care patients meet candidate eligibility criteria, how often FIB-4 can be calculated, the distribution of scores by age and metabolic risk, and the expected volume requiring second-line testing. This phase can be conducted using EHR data and would provide immediate service-planning information.

Phase 2: Validate FIB-4 against an appropriate reference. Prospective studies should compare FIB-4 with VCTE, ELF and, where clinically indicated, more definitive assessment. Particular attention should be paid to type 2 diabetes, older adults and younger high-risk adults. The objective is not necessarily to create new cut-offs, but to determine whether internationally used thresholds produce acceptable sensitivity, specificity and referral burden in Saudi populations.

Phase 3: Pilot an automated primary-care pathway. Selected primary-care centres could implement EHR-triggered FIB-4 with standardised second-line testing and referral. Outcomes should include uptake, missing laboratory components, proportion low/intermediate/high risk, VCTE completion, referral completion, newly detected advanced fibrosis, clinician acceptability, patient acceptability and time to specialist review.

Phase 4: Evaluate service impact and cost-effectiveness. A pragmatic implementation study should quantify changes in referral volume, unnecessary referral, VCTE demand, specialist workload, detection of advanced fibrosis and costs. Saudi-specific cost-utility modelling could then compare FIB-4/VCTE, FIB-4/ELF and existing practice.

Phase 5: Comparative effectiveness. Only after feasibility, diagnostic pathways and service capacity are established should a larger comparative study test whether systematic FIB-4-based case-finding improves clinically meaningful outcomes over usual care. Depending on feasibility, this could use cluster randomisation, stepped-wedge implementation or another pragmatic design, rather than assuming that an individually randomised trial is always the best approach.

This staged programme also provides continuity between earlier biomarker research and the current implementation question. The field has progressed from asking whether routinely available markers predict risk to asking how a risk-stratification pathway can be embedded in real health systems. Saudi research is now well positioned to address that second question. The proposed research priorities for evaluating and implementing FIB-4-based risk stratification in Saudi primary care are summarized in Table 3.

## 4. Discussion

The evidence now supports a clear conceptual shift in Saudi liver-disease detection. Earlier work established that MASLD is common and that simple non-invasive markers can distinguish differences in fibrosis risk. Contemporary international guidance has converted that evidence into multistep pathways, and recent Saudi studies have begun to provide the local epidemiological and clinical context needed for implementation.

FIB-4 is well suited to a first-line role because it is inexpensive, familiar and potentially automatable. Its limitations are precisely why it should be embedded in a pathway rather than promoted as a stand-alone test. The main risk of poorly designed implementation is not simply diagnostic inaccuracy; it is service distortion. If every FIB-4 result above a single threshold triggers direct specialist referral, false-positive results and age effects could create unnecessary workload. Conversely, if FIB-4 is only calculated after abnormal liver enzymes, patients with normal aminotransferases but important metabolic risk may remain undetected. Sequential testing offers a way to balance these competing problems.

The Saudi evidence is encouraging but still incomplete. The Alqahtani et al. cohort study provides high-quality contemporary evidence of disease burden and VCTE-defined fibrosis risk [12]. The Saudi consensus provides a locally adapted clinical framework [13]. The 2026 primary-care study demonstrates that FIB-4 can reveal risk in patients with normal liver enzymes who might otherwise be missed [14]. What is not yet established is the performance and system impact of a standardised FIB-4-first pathway across diverse Saudi primary-care settings.

This distinction matters for policy. Evidence that a test predicts fibrosis is necessary but not sufficient for a national programme. Implementation requires laboratory readiness, EHR logic, access to second-line testing, referral agreements, clinician education, patient communication and quality monitoring. It also requires an explicit definition of success. Relevant outcomes include not only diagnostic accuracy but also the proportion of eligible patients assessed, time to VCTE, proportion of unnecessary referrals, detection of advanced fibrosis, regional equity, cost and retention in follow-up.

The therapeutic landscape makes this work more urgent and changes the meaning of case-finding. The 2026 global consensus integrates FIB-4, VCTE and ELF with treatment initiation and response monitoring, reflecting the emergence of resmetirom and semaglutide as liver-directed options for selected non-cirrhotic MASH with F2–F3 fibrosis [15]. Primary care therefore sits at the beginning of a potentially actionable pathway. Treatment availability does not, however, justify indiscriminate screening, and therapeutic thresholds derived from international consensus should not be adopted as Saudi national thresholds without evaluation of local regulatory status, access, cost, specialist capacity and population performance.

This review has limitations. It is a narrative rather than a systematic review and therefore does not provide pooled estimates or a formal risk-of-bias assessment. The Saudi empirical literature on FIB-4 implementation remains small, and some recommendations necessarily draw on international evidence. The proposed framework should therefore be viewed as a structured hypothesis for implementation and research, not as a replacement for formal national guideline development.

## 5. Conclusions

Saudi Arabia now has a stronger evidence base for moving from recognition of MASLD burden toward structured fibrosis risk stratification in primary care. FIB-4 remains a practical first-line tool, but its value lies in its position within a sequential pathway rather than in stand-alone diagnosis. The September 2026 global consensus reinforces a FIB-4-first, VCTE/ELF-second approach and demonstrates that fibrosis assessment is increasingly linked not only to prognosis and referral but also to treatment eligibility and response monitoring [15]. Recent Saudi population data, local consensus recommendations and early real-world primary-care evidence make local implementation timely. The next step should not be uncritical adoption of an imported algorithm. Saudi primary care requires a locally evaluated pathway that defines the target population, tests performance in important subgroups such as type 2 diabetes, ensures laboratory and EHR readiness, links FIB-4 to accessible second-line testing and specialist care, and measures outcomes, equity and cost. A staged programme of validation, feasibility, implementation and comparative-effectiveness research can provide the evidence needed to move from risk prediction to sustainable, clinically actionable care.

## Figures and Tables

**Table 1:** Contemporary guidance relevant to FIB-4-based risk stratification in non-specialist settings.

Guidance	Target Population	First-Line	Low-Risk Approach	Second-Line/Referral	Key Implementation Point
AASLD 2023 [7]	Steatosis/suspected NAFLD; high-risk groups incl. T2DM and complicated obesity	FIB-4	<1.3: primary-care follow-up; repeat based on metabolic risk	≥1.3: VCTE/ELF or further risk stratification; >2.67 supports specialist referral	Age adjustment > 65 y; low accuracy < 35 y; normal aminotransferases do not exclude fibrosis
EASL-EASD-EASO 2024 [8]	T2D; abdominal obesity + ≥1 metabolic risk factor; persistent liver-enzyme elevation	FIB-4	<1.3 generally low risk; reassessment at intervals	Elastography second step; ELF alternative	Explicit multistep case-finding model
Saudi consensus 2025/26 [13]	Saudi patients at risk of MASLD/MASH	FIB-4 with other NITs	FIB-4 < 1.3: PCP follow-up	High-risk NIT results: hepatology referral; LSM used for intermediate/high risk	Local adaptation of international standards; implementation evidence still limited
AGA 2026 [10]	Patients at risk for clinically significant fibrosis	FIB-4-based pathway	Low-risk patients managed outside hepatology	Second-tier NITs and specialist escalation according to risk	Dedicated implementations focus across primary care and specialty settings

Abbreviations: AASLD, American Association for the Study of Liver Diseases; AGA, American Gastroenterological Association; ELF, Enhanced Liver Fibrosis test; LSM, liver stiffness measurement; NIT, non-invasive test; PCP, primary-care provider; T2DM, type 2 diabetes mellitus; VCTE, vibration-controlled transient elastography.

**Table 2:** Key Saudi evidence informing the proposed primary-care implementation agenda.

Study/Source	Design/Setting	Main Contribution	Strength	Limitation for Implementation
Alenezi et al., 2023 [11]	Systematic review/meta-analysis of Saudi NAFLD prevalence	Established earlier Saudi burden and major heterogeneity	National evidence synthesis	Predates MASLD era and recent VCTE-based population evidence
Saudi MASLD consensus [13]	Modified Delphi, multidisciplinary Saudi panel	Local recommendations for risk stratification, referral and management	Context-specific professional consensus	Not a real-world implementation or outcomes study
Zahrani et al., 2026 [14]	Retrospective, single-center primary care; *n* = 200 high-risk adults with normal liver enzymes	Demonstrated feasible FIB-4 stratification and low referral despite intermediate/high scores	Directly relevant to primary-care case-finding	Small, selected sample; no definitive fibrosis reference standard
Alqahtani et al., 2026 [12]	Prospective population-based cohort, 7 primary-care centers; *n* = 2979; VCTE	Adjusted MASLD prevalence 34.2%; significant fibrosis in 4.3% of MASLD	Large, contemporary, multicenter phenotyping	Does not by itself define the optimal FIB-4-first implementation pathway

**Table 3:** Proposed Saudi Research Roadmap for FIB-4-Based Risk Stratification in Primary Care.

1. Epidemiology/Readiness	2. Local Validation	3. Feasibility Pilot	4. Implementation & Economics	5. Comparative Effectiveness
Eligible population FIB-4 calculability risk distribution	FIB-4 vs. VCTE/ELF Age/T2DM subgroup referral thresholds	EHR automation workflow uptake patient/clinician acceptability	Referral burden cost-effectiveness regional access	Pragmatic trial clinical outcomes long-term sustainability

## Data Availability

No new data were created or analysed in this study. Data sharing is not applicable to this article.
